# Concentration of viruses and electron microscopy

**DOI:** 10.18699/VJ20.620

**Published:** 2020-05

**Authors:** I.D. Petrova, B.N. Zaitsev, O.S. Taranov

**Affiliations:** State Research Center of Virology and Biotechnology “Vector”, Rospotrebnadzor, Koltsovo, Novosibirsk region, Russia; State Research Center of Virology and Biotechnology “Vector”, Rospotrebnadzor, Koltsovo, Novosibirsk region, Russia; State Research Center of Virology and Biotechnology “Vector”, Rospotrebnadzor, Koltsovo, Novosibirsk region, Russia

**Keywords:** virus concentration, electron microscopy, virus purification, virus detection, review, концентрирование вирусов, электронная микроскопия, очистка вирусов, выявление вирусов, обзор.

## Abstract

Nearly all lethal viral outbreaks in the past two decades were caused by newly emerging viruses. Viruses are often studied by electron microscopy (EM), which provides new high-resolution data on the structure of viral particles relevant to both fundamental virology and practical pharmaceutical nanobiotechnology. Electron microscopy is also applied to ecological studies to detect viruses in the environment, to analysis of technological processes in the production of vaccines and other biotechnological components, and to diagnostics. Despite the advances in more sensitive methods, electron microscopy is still in active use for diagnostics. The main advantage of EM is the lack of specificity to any group of viruses, which allows working with unknown materials. However, the main limitation of the method is the relatively high detection limit (107 particles/mL), requiring viral material to be concentrated. There is no most effective universal method to concentrate viruses. Various combinations of methods and approaches are used depending on the virus and the goal. A modern virus concentration protocol involves precipitation, centrifugation, filtration, and chromatography. Here we describe the main concentrating techniques exemplified for different viruses. Effective elution techniques are required to disrupt the bonds between filter media and viruses in order to increase recovery. The paper reviews studies on unique traps, magnetic beads, and composite polyaniline and carbon nanotubes, including those of changeable size to concentrate viral particles. It also describes centrifugal concentrators to concentrate viruses on a polyethersulfone membrane. Our review suggests that the method to concentrate viruses and other nanoparticles should be chosen with regard to objectives of the study and the equipment status of the laboratory.

## Abstract (ru)

Почти все смертельные вирусные вспышки в последние два десятилетия были вызваны вновь появляющимися вирусами. Для изучения вирусов часто используют электронную микроскопию (ЭМ). Она позволяет получить новые данные о структуре вирусных частиц с высоким разрешением, что представляет интерес как для фундаментальной вирусологии, так и для практической фармацевтической нанобиотехнологии. Кроме того, ЭМ применяется в экологических исследованиях для определения наличия вирусов в окружающей среде, при анализе технологических процессов для производства вакцин и других биотехнологических компонентов, а также в диагностических целях. Несмотря на развитие более чувствительных методов, электронная микроскопия в диагностике остается рабочим методом. Главное преимущество ЭМ – отсутствие специфичности к какой-либо определенной группе вирусов, что способствует работе с неизвестным материалом. Однако основное ограничение метода – относительно высокий предел обнаружения (107 частиц/мл), в связи с чем необходимо концентрировать вирусный материал. Не существует какого-то одного наиболее эффективного метода. В зависимости от самого вируса и поставленной цели используются различные комбинации методов и подходов. В настоящее время концентрирование вируса включает операции осаждения, центрифугирования, фильтрации и хроматографии. В обзоре на примере разных вирусов описаны эти основные методы. Существует необходимость в разработке эффективных методик элюирования, которые могут нарушить связь между фильтрующими материалами и вирусами, чтобы повысить степень восстановления. Рассмотрены рабо ты по созданию уникальных ловушек, магнитных шариков, композитных полианилиновых и углеродных нанотрубок и нанотрубок с изменяемым размером для концентрирования вирусных частиц. Приведен пример применения центрифужных концентраторов, в которых вирус осаждается на мембране из поли- эфирсульфона. Проанализированные данные указывают на то, что способ концентрирования вирусов или других наночастиц выбирается в каждом конкретном случае в зависимости от поставленной цели и оснащенности лаборатории.

## Introduction

Electron microscopy (EM) is often used to study viruses. This method makes it possible to determine the shapes and sizes of viral particles and their localization within cells, as well as to identify cytological changes in cells. The main advantage of EM is the lack of specificity to any group of viruses, as opposed to immunological and molecular tests. With the present advances in genetic engineering and the use of a wide range of pseudovirus-based assays in vaccine development, the list of possible research targets for EM and the scope of problems solved have expanded significantly. A valid EM study requires that the sample should contain enough viral particles. It was shown earlier (Reid et al., 2003) that EM visualization requires virion contents no lower than 107 particles/mL. Thus, the necessity for purification and concentration of viral material arises rather often. It is noteworthy that EM is also necessary for the refinement of virus concentration methods. Moreover, in the age of nanotechnology we cannot ignore the fact that transmission EM (TEM) is used to control the fabrication of nanoparticles and their application (Zajtsev et al., 2016).

In addition to the classical application of the method, i. e. studying structures of viruses, infiltration processes, and virus transmission in organisms, EM is also used in at least three fields of applied research: diagnostic EM (DEM); ecological EM (detection of viruses in the environment), and problems associated with technological processes (manufacturing of vaccines and other components) that use biotechnological methods to yield the products and require that the product should be proven to be virus-free.

## Diagnostic electron microscopy

Despite the advances in more sensitive methods, such as polymerase chain reaction (PCR) or immunoenzyme analysis (IEA), EM still remains a widely applicable diagnostic tool (Goldsmith, Miller, 2009; Gentile, Gelderblom, 2014). The main advantage of EM in diagnostics of infectious diseases is its versatility, i. e. the ability to observe ‘anything’ in a single sample without prior knowledge of possible microorganism identities present in the sample (Hazelton, Gelderblom, 2003). Another advantage of EM is its fastness, as it takes as little as 10–15 minutes for a wide range of object sizes to be studied. EM makes it possible to analyze both liquid and tissue samples and requires no additional information, unlike the diagnostic methods using nucleic acids (PCR) or antibodies (IEA).

The main limitation of DEM is the virion content in samples. To overcome this limitation, various concentration techniques are used, such as ultracentrifugation, ultrafiltration, examination of ultrathin sections, etc. In (Beniac et al., 2014), the authors describe virus concentration by filtration straight onto EM support grid with holey carbon substrate Quantifoil R1/4 (Quantifoil Micro Tools GmbH, Großlöbichau, Germany). This technique combined with scanning EM (SEM) improves the virus detection threshold to contents of 102 viral particles per 1 mL.

Thus, DEM is a valuable method for observing newly emerging diseases and potential bioterrorism agents. Finally, ultrastructural studies performed using EM provide detailed examination of virus morphogenesis, virus – cell interactions, and pathogenetic aspects of viral infections including the development of preventives and treatments.

## Electron microscopy in ecological studies

The use of EM for ecological purposes (detection of viruses in the environment) appears to be an obvious solution, but it is rarely used in practice. When it is, its most common target is the pollution of water resources, which supplies almost illimited volumes of research material. One of the few papers dealing with seawater as a research subject was published in 1999 (Alonso et al., 1999). The described concentration process included two stages: concentration of a vast amount of seawater using a tangential flow filtration system and ultrafiltration using a centrifugal concentrator with further visualization by means of TEM. The same methods were used later in (Sun et al., 2014). The authors studied over 150 L of seawater from the Yangshan Deep- Water Port area (South East Shanghai, China). However, these days EM is quite rarely used for such analytical tasks, as they are mostly solved by more sensitive methods, first of all, PCR. The reason is the limited range of possible targets, which are being typically presupposed or known beforehand, so PCR is often substituted for EM at the stage when an infectious agent has to be detected.

## Analysis of biotechnological processes

Vaccines are among the most effective pharmaceutical products in public healthcare showing great results in terms of safety. Since biological materials are involved in vaccine production, the process must be protected from sporadical contamination. The necessity for controlling biotechnologically produced materials is broadly discussed (Sheets, 2013). The production of virus vaccines and other cellular biopharmaceutical products is a complex technological process, which involves various biological materials (e. g., various cell substrates, such as chicken eggs with embryos; primary cell cultures; and continuous cell lines). The whole process is potentially vulnerable to contamination by foreign agents that may be unintentionally brought in with the materials used or from environmental sources. An example of this type of study may be found in (Reid et al., 2003), where three methods for retrovirus contamination assessment in mouse culture supernatants and СНО cell lines were compared: direct count of viral particles in a solution with latex beads (detection limit 10^7^ particles/ mL), ultracentrifuge concentration in a discontinuous sucrose density gradient with EM (10^5^ particles/mL), and the method involving a thin section of a homogenous matrix of viral particles, cell debris, agar medium (sensitivity 10^5^ particles/mL). Routine EM is obviously not suitable for registering virus contamination.

Various biotechnological fields, such as development of vaccines and adjuvants, manufacturing of biologically active complexes, development of contrast media, targeted delivery, and microelectronics use virus-like particles studied with EM. For instance, preparation of spherical particles (SP) from tobacco mosaic virus was controlled by TEM. It was shown that SP could be visualized via TEM without contrasting agents (Trifonova et al., 2015).

## Virus concentration methods

There is no most effective universal method to concentrate viruses. Various combinations of methods and approaches are used depending on the virus and the problem set. Virus concentration processes currently include sedimentation, centrifugation, filtration, and chromatography (Transfiguracion et al., 2007; Vicente et al., 2011).

## Precipitation

Virus precipitation is commonly achieved with polyethylene glycol (PEG), ammonium sulfate, or calcium phosphate. It is a convenient tool for obtaining viruses in both large and small quantities, while also providing the advantage of virus separation from most heterologous proteins. For example, the precipitation and purification method using PEG 6000 with further ultracentrifugation and clarification widely used for influenza viruses was successfully applied for concentration and purification of avian influenza Type A virus (ten different strains) from virus-containing suspensions (Ismagambetov et al., 2017). Another paper (Ryabinnikova et al., 2015) presented data on purification and concentration of horse rhinopneumonia (HRP) virus using various techniques. It was found that concentration of HRP virus using PEG-6000 with 0.5 М sodium chloride with subsequent dialysis yields concentrates with high infectious and antigen activity. It was shown that adsorption in presence of PEG produced virus preparations suitable for manufacturing inactivated HRP vaccine.

Polyethylene polyamine (PEPA) with a mass of 16,000 D was shown to be more suitable for concentration of FMD virus and porcine enterovirus (serotype 7) by precipitation than PEG-6000 (Bahutashvili et al., 2002). It was found that the most complete precipitation of FMD virus was achieved at PEPA contents of 0.1 and 0.2 %. There, virus losses were 0.74 and 0.11 %, respectively. It was also shown that vaccines produced using the preparations obtained via two different precipitation techniques were different in terms of their efficacy. The PEPA-based technique was preferable since the respective vaccine was three times as effective.

## Centrifugation and density gradient

Centrifugation is an easy-to-use large-scale separation method based on differences in density. Due to small sizes of viral particles, high speed and centrifugal force are required, which, however, can make the particles noninfectious (Burova, Loffe, 2005). Centrifugation in density gradient using sucrose, cesium chloride (CsCl), or iodixanol is a viable alternative. The use of sucrose provides a viscous hyperosmotic solution. Density gradient of iodixanol is a low-viscosity system capable of producing isotonic solution and maintaining virus functionality (Gias et al., 2008). It was shown (Segura et al., 2006) that the use of iodixanol gradient provides 37 % retrovirus recovery with a promising purity of 95 %. To separate horse influenza virus from virus-containing allantoic fluid, ultracentrifugation in sucrose density gradient and DEAE cellulose-based ion exchange chromatography were used (Tajlakova et al., 2011). It was found that complete virus adsorption by ion exchanger occurred in a buffer solution with рН 7.0, whereas elution required a buffer with 0.5 М sodium chloride solution рН 7.4. As a result, virus preparations were obtained with ballast protein removal of 98 %.

## Filtration

To concentrate enteroviruses from various water bodies, a unified membrane filtration method was developed using the microfiltration (MF) mode of the MFM 0142 module equipped with MMK1 membrane (Sanamyan et al., 2006). The method demonstrated high efficacy in enterovirus concentration from water with various degrees of contamination (potable, underground, river, and waste within the permissible limits) with the concentration time shortened to 42 min. The MMK-type filtration membrane modified with 0.5 % amino compounds proved most effective among the studied membranes. Hollow fiber MF membranes (d = 0.2 μm) were used to study the process of human adenovirus 2 (HAdV-2) concentration (Lu et al., 2016) depending on the virus content varying between 1.3 × 10^7^ and 3.4 × 10^8^ copies/mL.

To optimize the separation of hepatitis А virus from water, membranes based on polyamide, cellulose nitrate, cellulose acetate, and polyethersulfone were studied (Zalesskikh, Bystrova, 2018). Glass fiber, cardboard, and polypropylene were used as pre-filters. It was shown that the most effective membrane combination for filtration of highly impure water includes a cardboard or polypropylene filter for preliminary filtration and a polyamide filter for the main stage of the process. A pressure filtration device (AF-142K, Vladisart, Vladimir, Russia) was used for filtration of samples with water volumes up to 10 L carried out at the pressure of 2 atm.

Epidemiological surveillance over human enteroviruses requires virus concentration from wastewater. This typically involves processing of large volumes (100–1000 L) of water, the most common method being the VIRADEL process with microporous filters. Positively charged filters do not require sample pretreatment, and they can concentrate viruses from water with a wider pH range than electronegative filters due to the virus surface charge typically being negative. Virosorb 1MDS is one of the most common electropositive filters. In recent years, it has been joined by positively charged NanoCeram filters (Soto-Beltran et al., 2013). Virus elution from filters after concentration was performed using organic (beef extract) or inorganic (sodium polyphosphates) solutions. Then, the eluates were repeatedly concentrated to reduce sample volumes and improve virus detection. Most filters proved highly effective in capturing the virus, while the elution and repeated concentration methods showed varying degrees of success due to the biological variation of viruses present in water (Ikner et al., 2012).

Nanofiltration (NF) was used in the filtration of serum protein solutions, production of serum derivatives, and manufacturing of a wide range of biopharmaceutical products. Depending on the material used, NF membranes fall into two main groups: ceramic and polymeric ones. Hybrid NF membranes are also of interest due to their performance under suboptimal conditions. Various polymers, such as polyethersulfone (Maximous et al., 2009), polydimethylsiloxane (Yousefi et al., 2017), polyvinylidene fluoride (Dong et al., 2013), and polysulfone (Ghaee et al., 2017) were tested as top layer materials for hybrid membranes. The use of polymers for obtaining hybrid NF membranes makes it possible to change pore sizes, chemical properties, and charges of membrane surfaces. Nanofiltration increasingly becomes a common step in pharmaceutical production, since it does not raise toxicity issues (Doodeji, Zerafat, 2018). Obtaining a purified virus concentrate is one of the key steps in vaccine production. Polyethersulfone membrane 300 kD combined with weak anionic detergent solution appears to be the most suitable for influenza virus concentration and removal of small molecule impurities (Kyzin et al., 2014).

Elution and repeated concentration methods were reported in the literature (Falman et al., 2019). First, wastewater was concentrated with ViroCap cartridge filters, and then Celite beef extract, ViroCap flat disc filters, InnovaPrep concentrating pipettes, PEG/NaCl precipitation, and skimmed milk flocculation were used. PEG/NaCl sedimentation and skimmed milk flocculation turned out to be the most effective methods among the five methods tested in experiments with poliovirus Type 1 (PV 1). Optimization of the skimmed milk flocculation method increased PV 1 recovery nearly twofold as compared to PEG/NaCl precipitation.

Tangential flow filtration is a common method of filtration by size (Wickramasinghe et al., 2005). Influenza virus was used to test a two-step purification and concentration protocol for viral particles.

## Chromatography

Chromatography is a separation method based on the interaction between the target virus and column matrix. In general, separation functionality depends on charge, size, hydropathicity, and affinity.

Ion exchange chromatography utilizes the phenomenon of ion exchange between an immobile solid phase and a mobile liquid phase, which makes it possible to separate structurally and morphologically similar viral particles provided they have different total charges and/or charge distributions over their outer surfaces (Ruščić et al., 2015). Virus elution may require different salt concentrations, and the method can be applied to any mixed infection if viruses have different isoelectric points. A simple separation and concentration technique for mixed infection by cowpea chlorotic mottle virus (CCMV) and cucumber mosaic virus (CMV) is proposed in (Ali, Roossinck, 2008).

Monolithic supports made it possible to use chromatography for purification and concentration of various viruses (Svec et al., 2011; Krajačić et al., 2017). Unlike the classical chromatographic supports with diffusion-based mass transfer and relatively small pores, monoliths are characterized by significantly enhanced mass transfer due to convection and channel sizes of several microns. I. Gutiérrez-Aguirre et al. (2009) showed that CIM QA supports effectively retained rotaviruses present in stool samples, as well as in wastewater and river water samples. Concentration of rotaviruses was achieved by elution of the retained viruses with 1 М NaCl solution. The obtained viruses preserved their integrity, as confirmed by electron microscopy.

Size exclusion chromatography (or gel filtration in case of aqueous liquid phase) is a fluid chromatography based on varying capabilities of different-sized molecules to infiltrate the pores of non-ionogenic gel acting as a stationary phase. In this separation method, a matrix of densely packed silica gel or agarose gel is used (Barth et al., 1994). The main advantages of this method are its simplicity and the low cost of resins. Nevertheless, the method suffers from the lack of selectivity, requires low flow velocity, and shows low overall performance. Tick-borne encephalitis virus was used to refine the gel filtration method for the Superdex 200 column (Havlik et al., 2014). The samples obtained were tested by the following methods: (1) the immunological (dot blot) technique for testing biological activity, (2) gasphase electrophoretic mobility macromolecular analysis (GEMMA) for determining particle sizes, and (3) atomic force microscopy (AFM) or TEM for obtaining information on shapes and sizes of viral particles. The mean diameter of inactivated tick-borne encephalitis viral particles determined using GEMMA was 46.6 ± 0.5 nm, as opposed to AFM and TEM images, which showed the diameters of about 58 ± 4 and 52 ± 5 nm, respectively.

Hydrophobic interaction chromatography is based on particle adsorption at weakly hydrophobic surface at high salt contents with subsequent elution with descending salt gradient. Virus surfaces include hydrophobic areas, which are linked to immobilized hydrophobic ligands at chromatographic carriers (Roettger et al., 1989). The limitation of the method is illustrated by an example with adenovirus (Schagen et al., 2000). It was found that large salt concentrations could not only reduce the virus immunogenicity but could also favor viral particle aggregation.

Polyvinyl alcohol cryogel with pore sizes of 0.04–2.0 μm was used in a concentration method with virus capturing by bioaffine sorbent (Lozinskiy et al., 2013). The use of the sorbent was tested in the concentration process for influenza Type А viruses H1N1 and H3N2, parainfluenza virus type 6, smallpox virus, and FMD viruses. Bioaffine sorbent based on viscoelastic, non-brittle, and hydrolytically stable PVA cryogel allows virus concentration not only in a chromatographic column, but in reactors with sorbent mixing, which significantly intensifies mass transfer. The advantage of the method is that it provides the possibility of concentration not only for small but also for the largest (0.5 μm and above) viruses. According to the invention description, the bioaffine sorbent may include not only antibodies but also immobilized enzymes capable of modifying the virus.

## Other methods

To monitor the presence of influenza virus in open water bodies, a custom device was designed (Levchenko et al., 2007). It employed magnetic immunosorbents in which antibodies were immobilized on a solid magnetic support.

Sorption of influenza viruses onto polyaniline (PANI) and carbon nanotubes, as well as on PANI composites (nanotubes and grains) with or without silver, was studied (Ivanova et al., 2015). It was found that inclusion of silver increased the sorption capability of PANI tubes in case of allantoic influenza Type А viruses. PANI nanotube composites with 30 % silver appear to be the most promising virus sorption material in water solutions with regard to the combination of properties.

Hepatitis А virus (HAV) was used as a model for comparison of various carbohydrate-binding lectins, including concanavalin А (Con A), wheat germ agglutinin, and soybean agglutinin, based on their binding affinity to the virus (Ko et al., 2018). Con A showed higher binding affinity than other lectins. Con A-bound immunomagnetic separation combined with RT-PCR made it possible to detect HAV at 10–4 dilution of the initial virus concentration (the titer being equal to 104 infectious doses for cell culture per mL). It shows that Con A may be a promising candidate for HAV concentration.

It was shown in several papers (Flavigny et al., 2004; Sakudo et al., 2009b) that magnetic beads coated with anionic polymers, such as poly-(methyl vinyl ether-co-maleic anhydride) may be used for effective capture of various types of viruses, which include human immunodeficiency virus Type 1 (Sakudo, Ikuta, 2012), respiratory-syncytial virus (Sakudo et al., 2009a), Borna disease virus (Sakudo et al., 2011b), influenza virus (Sakudo et al., 2008), and Dengue fever virus (Sakudo et al., 2011a). The method was later applied to adenovirus as well (Sakudo et al., 2016).

Japanese researchers (Mogi et al., 2016) worked on a new virus concentration method. They proposed and experimentally demonstrated a virus concentration device, which employed the ion depletion zone generated by ion polarization. The efficacy was assessed with fluorescent nanoparticles, Baculovirus, albumin, and dextran. All samples were successfully concentrated.

A new virus concentration strategy using the expression of human poliovirus receptor gene (hPVR) on Escherichia coli cell surfaces was developed (Abbaszadegan et al., 2011). The ability of modified bacterial cells to capture viral particles was confirmed by TEM. This approach provides new opportunities for effective capture and concentration of water-transmitted viruses.

To process field samples, unique microdevices capable of effective virus enrichment and concentration were designed using carbon nanotubes with variable sizes (CNT-STEM) (Yeh et al., 2016). The intertube distance between CNTs could be set between 17 and 325 nm to precisely match the sizes of specific viruses. With this device, the authors managed to identify two new strains.

The paper recently published by B.N. Zajtsev et al. (2019) described the use of a Vivaspin centrifugal concentrator. Viral particles were concentrated on a double vertical polyethersulfone membrane. Following centrifugation, the membrane was retrieved from the concentrator to assess the number of particles sedimented on the membrane by EM examination of ultrathin sections.

## Conclusions

The desired research goal plays a major part in choosing the virus concentration method. For instance, ecological, pharmaceutical, and medical problems require different approaches. For a water contamination test to be successful, one should filter hundreds of liters of water from a specific area and then collect the virus material sedimented on the surface and inside charged filters. This procedure is typically followed by the second filtration step using different filters with adjusted pore sizes (Tarasov et al., 2012). A series of gains have been achieved in designing materials for filtration membranes in recent years. However, much is to be done for complete virus recovery from membranes and membrane surfaces.

To improve the virus concentration technology, one should focus on surface interactions between viruses and filter materials. In particular, effective elution methods should be developed to disrupt the bonds between filter materials and viruses to increase recovery. In diagnostic studies in specific patients, the amount of material is often limited to few milliliters. In many cases, especially when it comes to mixed infections, electron microscopy turns out to be one of the most reliable virus detection tools. Certainly, ultrathin sections can only be obtained at wellequipped EM laboratories, where EM diagnostics of virus infections is a routine process.

## Conflict of interest

The authors declare no conflict of interest.
